# Antitumor activity against murine lymphoma L5178Y model of proteins from cacao (*Theobroma cacao *L.) seeds in relation with *in vitro *antioxidant activity

**DOI:** 10.1186/1472-6882-10-61

**Published:** 2010-10-20

**Authors:** Ana M Preza, María E Jaramillo, Ana M Puebla, Juan C Mateos, Rodolfo Hernández, Eugenia Lugo

**Affiliations:** 1 Departamento de Graduados e Investigación en Alimentos, Escuela Nacional de Ciencias Biológicas, Instituto Politécnico Nacional, Carpio y Plan de Ayala S/N, Delegación Miguel Hidalgo, 06470 México, D.F., México; 2 Laboratorio de Inmunofarmacología de Productos Naturales, Centro de Investigación Biomédica de Occidente, I.M.S.S., Sierra Mojada No. 800, Col. Independencia, 44340 Guadalajara, Jalisco, México; 3 Centro de Investigación y Asistencia en Tecnología y Diseño del Estado de Jalisco, A.C., Av. Normalistas 800, Colinas de la Normal, 44270 Guadalajara, Jalisco, México

## Abstract

**Background:**

Recently, proteins and peptides have become an added value to foodstuffs due to new knowledge about its structural analyses as related to antioxidant and anticancer activity. Our goal was to evaluate if protein fractions from cacao seeds show antitumor activity on lymphoma murine L5178Y model. The antioxidant activity of these fractions was also evaluated with the aim of finding a correlation with the antitumor activity.

**Methods:**

Differential extraction of proteins from unfermented and semi-fermented-dry cacao seeds was performed and characterized by SDS-PAGE and FPLC size-exclusion chromatography. Antitumor activity was evaluated against murine lymphoma L5178Y in BALB/c mice (6 × 10^4 ^cells i.p.), with a treatment oral dose of 25 mg/kg/day of each protein fraction, over a period of 15 days. Antioxidant activity was evaluated by the ABTS^+ ^and ORAC-FL assays.

**Results:**

Albumin, globulin and glutelin fractions from both cacao seed type were obtained by differential solubility extraction. Glutelins were the predominant fraction. In the albumin fraction, polypeptides of 42.3 and 8.5 kDa were found in native conditions, presumably in the form of two peptide chains of 21.5 kDa each one. The globulin fraction presented polypeptides of 86 and 57 kDa in unfermented cacao seed that produced the specific-cacao aroma precursors, and after fermentation the polypeptides were of 45 and 39 kDa. The glutelin fraction presented proteins >200 kDa and globulins components <100 KDa in lesser proportion. Regarding the semifermented-dry cacao seed, it was observed that the albumin fraction showed antitumoral activity, since it caused significant decreases (p < 0.05) in the ascetic fluid volume and packed cell volume, inhibiting cell growth in 59.98 ± 13.6% at 60% of the population; while the greatest antioxidant capacity due to free radical scavenging capacity was showed by the albumin and glutelin fraction in both methods assayed.

**Conclusion:**

This study is the first report on the biological activity of semifermented-dry cacao protein fractions with their identification, supporting the traditional use of the plant. The albumin fraction showed antitumor and free radical scavenging capacity, however both activities were not correlated. The protein fractions could be considered as source of potential antitumor peptides.

## Background

In living systems, as a part of metabolism pathways, free radicals are generated on an ongoing basis. Free radicals in biological systems include reactive oxygen species (ROS) such as O_2_^.-^, HO_2_^, ^H_2_O_2 _and OH^. ^and reactive nitrogen species (RNS). When free radicals are generated in excess or when cellular defenses are deficient, biomolecules are damaged by oxidative stress process. This process cause significant damage to tissues and biomolecules, providing conditions for the development of degenerative diseases. Due to the negative consequences of the oxidative processes, their inhibition both inside the organism and in foods is important, and is an area of very intense research. In fact epidemiologic surveys indicate that the diet may be related to 50% of all cancers in humans, because it may alter individual organ susceptibility and responsiveness to neoplastic processes, and therefore an increased demand in consumption of natural antioxidants from dietary supplements and traditional medicines is found [1]. Recently, proteins and peptides have become an added value to foodstuffs due to the new knowledge about their functional and biological activities. Many protein and protein hydrolysates also have been tested and found to have comparatively strong antioxidant activities, such as soy proteins, caseins and egg-yolk protein. The antioxidant activities of these proteins were directly related to their sulfhydryl (SH) content, amino acid residues, or their antioxidase activity (such as SOD, CAT, POD, GSH-Px) [2-6].

To understand how phytochemicals, such as proteins and peptides derived from whole foods interact with each other, specialized cancer models that measure the effect on the biochemical target are needed, including evidence of a potentially useful effect on tumor growth or progression. The development of *in vitro *screening procedures as tools to identify chemopreventive foods or their chemopreventive fractions is important in identifying potentially beneficial foods or diets best suited for cancer prevention [7,8]. The measurement of antioxidant activity is an important screening method to compare the oxidation/reduction potentials of phytochemicals in various systems to predict *in vivo *activity. Many chemical methods are currently in wide use, including the oxygen radical absorbance capacity (ORAC) assay, which is one of the tests developed to measure the total antioxidant capacities of biological samples and the only method so far that combines both inhibition time and degree of inhibition into a single quantity against the peroxyl radical, which is one of the most common ROS found in human biology [9-11].

Cacao (*Theobroma cacao *L.; family Sterculiaceae) is an ancient American crop, originated in the regions of central, southern and southeastern Mexico. Currently, the Forastero variety is established as a crop in the Mexican states of Chiapas and Tabasco, it is vigorous and resistant to diseases, with low nutrient demand for its growth conditions and a high yield, as opposed to the Criollo variety [12,13]. The seeds contain different types of bioactive compounds e.g. fat, alkaloids, polyphenols, flavanols, and procyanidins with health-promoting properties that have been thoroughly investigated. Regarding the antioxidant properties of cacao, such activity has been attributed to polyphenolic compounds present in the seed [14-16]. However, research into whether the cacao seeds contain other antioxidant components is scarce. The protein content of the cacao seed is 10 - 15% of the dry weight, with an amino acid profile rich in lysine, arginine, serine, proline, alanine, leucine, valine, phenylalanine, and methionine [17,18], which allows to elucidate the possibility of obtaining bioactive peptides.

Recent research works that analyzed the peptide structure of soy, reported the presence of hydrophobic amino acids in its N-terminal, such as valine or leucine, as well as histidine, methionine, tryptophane, proline and tyrosine residues, which sequence showed antioxidant capacity by metal chelating and free radical scavenging [19-22]. On the other hand, the presence of proline residues, lysine or arginine have also shown anticancer activity, where the Lunasin is a unique 43 amino acid long peptide that contains 8 Asp (D) residues in its C-terminal, an Arg-Gly-Asp (RGD) cell adhesion motif, a helix with structural homology to chromatin-binding proteins, as well as a novel 4.8 kDa peptide whose cancer preventive properties have been demonstrated in a mammalian cell culture model and in a skin cancer mouse model against chemical carcinogens, oncogenes, and inactivators of tumor suppressor proteins. Lunasin was found in the 2S albumin storage protein of soybean, it has recently been isolated from barley, wheat and amaranth (the glutelin fraction had the highest lunasin concentration), suggesting the possibility that lunasin or lunasin-like compounds could be found in other seeds [23,24].

Therefore, to evaluate functional compounds from natural sources that can be used for chemoprevention in large-scale animal studies and ultimately form human subjects, oral administration is an important feature of an ideal cancer-preventive agent to evaluate the capacity of a compound to be absorbed and to reach the target tissue in a bioactive state [23,25].

In this work our goal was to evaluate the presence of protein fractions with antitumor activity from seeds of *Theobroma cacao *L. employing lymphoma murine L5178Y as a model. In addition, the antioxidant activity of these fractions was also evaluated to try to find a correlation between antitumor and antioxidant activity.

## Methods

### *Theobroma cacao *L. protein fractionation

Cacao seeds of the Forastero variety were provided by the Finca Irlanda producers, from the Soconusco region, in the State of Chiapas, Mexico. Two types of seeds were used for their evaluation: unfermented seeds (UF) were taken from the pods, the mucilage and coat were removed together, and the seed kernel lyophilized and stored at -20°C. Semi-fermented and sun-dried (SFD) unroasted cacao seeds were obtained after one day of fermentation in jute bags, followed by a wash with water to eliminate the remaining mucilage, and then sun-dried. The coats were then removed and the seed kernel ground with an electrical mill and stored at -20°C. Proteins were extracted and fractioned according to solubility class using the procedure described by Voigt and Biehl [26] with some modifications. First, to avoid an irreversible denaturation of storage proteins by oxidation products of polyphenols during extraction, the acetone dry powder (AcDP) was prepared. The powdered seed kernel was defatted with petroleum ether for 24 h and then purine alkaloids were partially extracted with chloroform for 8 h. Afterwards, the polyphenols were extracted three times with an 80% (v/v) cold aqueous acetone containing 5 mM sodium ascorbate; subsequently with 70% (v/v) cold aqueous acetone; and finally the residual water was removed from acetone pellet by dehydration with 100% cold acetone. The AcDP obtained was evaporated to dryness in the fume cupboard overnight to yield a light orange solid (38.77%). The dry AcDP was extracted successively with 10 mM Tris-HCl (pH 7.5 containing 2 mM EDTA), 0.5 M NaCl (containing 2 mM EDTA and 10 mM Tris-HCl pH 7.5), 70% (v/v) ethanol and 0.1 N NaOH, to obtain the albumin, globulin, prolamin and glutelin fractions, respectively. All these solvents contained 5 mM sodium ascorbate. Each protein fraction was dialyzed against deionized water for 48 h at 4°C, with a change of solution every 24 h. Finally, the solutions were lyophilized and stored at -20°C. Protein concentrations were determined by the method of Lowry *et al. *[27] using bovine serum albumin as the standard.

### Animals

Male BALB/c mice (6 to 8 weeks old, 20 - 24 g) were provided by the Centro de Investigación Biomédica de Occidente (CIBO-IMSS). The animals were maintained in a specific pathogen free animal care facility, in regulated environment (22 ± 1°C, 50 - 60% relative humidity and 12-hour light cycle). All mice were fed a commercial diet (Lab Chow Purina-Mexico) with an autoclaved tap water *ad libitum. *For this study all procedures involving animals were performed according to protocols approved by the Research Center's animal care committee and in compliance with the National guidelines on animal welfare (NOM-062-ZOO-1999) [28].

### Sodium dodecyl sulfate-polyacrylamide gel electrophoresis (SDS-PAGE)

Electrophoresis was carried out using the Mini-PROTEAN Treta Cell system (BioRad Laboratories) with a 4% polyacrylamide stacking gel and a 12.0% polyacrylamide resolving gel in the presence of sodium dodecyl sulfate (SDS) using standard Tris glycine buffers [29]. The protein sample (1 mg/mL) was dissolved in buffer 20 mM Tris-HCl, 150 mM NaCl, pH 7,2 and mixed with a solubilization buffer Trizma base (pH 6.8) which contains 0.12 M SDS, 2 M glycerol, a pinch of bromophenol blue and reduced with 10% (v/v) 2-mercaptoethanol in boiling water for 5 min. The protein sample was then loaded onto each well and the stacking and resolving gels were run in succession at 60 and 120 V, respectively, by a Bio-Rad electrophoresis constant power supply unit (Model 164-5052, Bio-Rad Laboratories). After electrophoresis, gels were gently agitated overnight in a fixing solution with 0.05% solution of Coomassie brilliant blue R250 in 50% (v/v) methanol and 10% (v/v) acetic acid. It was then treated with developer (40% (v/v) methanol and 10% (v/v) acetic acid) until the bands came out. Molecular masses were determined using a commercial broad-range protein molecular mass marker and analyzed with the help of software Quantity-One (BioRad Laboratories).

### FPLC size-exclusion chromatography

Protein fractions were dissolved in buffer: 20 mM Tris-HCl, 150 mM NaCl, pH 7.2 for albumin and globulin fraction; 70% (v/v) ethanol, 150 mM NaCl for prolamin fraction and 20 mM potassium phosphate, 1.0 M NaCl containing 1% (v/v) NLS for glutelin fraction. 2 mg/mL of the suspensions from each fraction were membrane filtered (0.45 μm mean pore size) and analyzed on a GE Healthcare, FPLC system, equipped with molecular exclusion Superdex columns (200 HiLoad 16/60 with exclusion range: 10 - 600 KDa and G75 HR 10/30 GL with exclusion range: 3 - 70 KDa) at 18°C, an UV detector fixed at 280 nm and a fraction collector. Data acquisition and processing were performed using the FPLC director software. The columns were equilibrated and eluted with the same buffer mentioned above. The flow rate was maintained at 1 mL/min collecting 1 mL fractions. Polypeptides were detected by absorbance at 280 nm. Fractions corresponding to each peak were collected and stored at -20°C until analysis. The columns were calibrated by using the protein molecular weight kit MW-GF-1000 (Sigma^®^).

### Murine model of lymphoma L5178Y and antitumor activity

The mouse model was used to evaluate if oral administration of proteins fractions from cacao could protect against the development of cancer. A lymphoma L5178Y cell line was used, derived from a thymic lineage (haplotype H-2d) tumor induced in a DBA/2 mouse by methylcholanthrene adapted to an ascitic form, and maintained by intraperitoneal (i.p.) transplantation of 10 × 10^6 ^cells/mouse every 15 days in syngenic BALB/c mice [30,31]. The murine model of lymphoma L5178Y is described as follows. On day 0, BALB/c mice were inoculated i.p. with 0.1 mL of fresh ascetic fluid (6 × 10^4 ^cells/mouse suspended in isotonic saline solution [0.9% NaCl w/v]). After 24 h, they were randomized into 5 groups of 5 mice each. A control group was injected with tumor cells but treated with 0.1 mL of isotonic saline solution only, and four groups were injected with tumor cells and individual treated with globulin UF, glutelin UF, albumin SFD and glutelin SFD (0.1 mL at non-lethal oral dose of 25 mg/kg/day dissolved in isotonic saline solution). On day 17, the mice were euthanized by ethyl ether inhalation, and ascites fluid and tissues were removed. Antitumor activity of protein fractions was measured according to the following parameters: 1) Body weights recorded at day 0 and once per week for 2 consecutive weeks; 2) ascites fluid collected from the peritoneal cavity, with volume measured in a graduated centrifuge tube and packed cell recovered by centrifugation at 2500 rpm for 10 min; and 3) tumor cell count determined by ascites fluid diluted ten-fold with isotonic saline solution, with viable cells counted (Trypan Blue exclusion) on the Neubauer counting chamber (Tumoral cells/mL ascites fluid). As an alternative measure of disease in the mice, spleen weights were also recorded to observe splenomegaly patterns [32].

### Antioxidant activity as free radical scavenging capacity

Two methods were used to compare the antioxidant activity of protein fractions from cacao.

The ABTS radical cation discoloration assay was adapted to microplates [33]. The ABTS radical cation (ABTS^+^) was activated by the reaction of 7 mM ABTS chemical with 2.5 mM potassium persulfate (both dissolved in deionized water) in the dark at room temperature for 12 - 16 h before use. Note that the resulting cation radical is stable in the dark at -74°C for 1 month or at 4°C for 1 week. For the study the ABTS^+ ^solution was diluted with PBS pH 7.4 or ethanol (~1:30), to an absorbance of 0.70 ± 0.02 at 690 nm. After dilution, Trolox standards (final concentration 0 - 600 μM) or samples (protein fraction 300 - 3000 ppm) in PBS or ethanol were added to each well. Then a diluted ABTS^+ ^solution was added to each well of the microplate (total volume of 290 μL) and the absorbance was monitored for 90 min at intervals of 15 min (the second reading at 6 min). Appropriate solvent blanks were run in each assay, while GHS was used as positive control (final concentration 10 - 300 μM). The discoloration of ABTS^+ ^was determined through a reduction in absorbance, which was a function of antioxidant concentration and equated to the reactivity of Trolox. Trolox equivalent antioxidant capacity (TEAC) for each sample was then calculated as mmols of Trolox equivalent per gram of protein fraction. Scavenging% was plotted as a function of the mass of protein contained in the sample in order to obtain the mass of protein necessary to reduce absorbance by 50% (IC_50_).

An ORAC method using fluorescein (FL) as the "fluorescent probe" has been developed [34]. The automated ORAC assay was carried out on a Tecan Safire plate reader controlled by the Magellan software with fluorescence filters for an excitation wavelength of 485 nm and an emission wavelength of 538 nm. The measurements were made in 96-well black flat bottom microplates. The reaction was performed at 37°C as the reaction was started by thermal decomposition of AAPH in 75 mM phosphate buffer (pH 7.4). In each well, sample (protein fraction 10 - 300 ppm), positive control (GHS) (final concentration 10 - 300 μM), blank (PBS) or standard (Trolox 0 - 80 μM) and FL (72 nM final concentration) were pipetted, and then the microplate was put into the pre-warmed plate (37°C). Then AAPH (12 mM final concentration) were added using an 8-channel pipette. The fluorescence was measured immediately every minute for a kinetic cycle of 80 min. The final results were calculated by monitoring the fluorescence decay curve of FL. The area under the fluorescence decay curve (AUC) was then integrated, and the net AUC calculated by the differences of areas under the fluorescent decay curve between the blank and the sample (expressed as μM Trolox/mg protein).

### Statistical analysis

The studies were carried out in single factor, using complete randomized design with two replications. The experimental results were expressed as the mean ± S.E.M. Data were assessed by one-way analysis of variance (ANOVA) and differences between samples were determined by LSD test; *P *value of < 0.05 was considered as statistically significant (STATISTICA for Windows version 4.3).

## Results and discussion

### Protein fractionation of cacao seeds

Proteins were extracted and fractionated according to their solubility characteristics; the ratio of UF and SFD cacao protein fractions is shown in Table 1. The same protein profile was observed in both types of seeds: % glutelin > % albumin ≥ % globulin > % prolamin. In this case, the prolamin and glutelin fractions were found, which is surprising since they have not been reported in previous works. Thus results cannot be compared with those described for cacao protein fractions under this same fractionation methodology [26,35,36]. Nevertheless, the SFD cacao seed showed a significantly higher protein content (p < 0.05), confirming that the content of total nitrogen in protein increased during the first day of cacao fermentation [35].

**Table 1:** Ratio of protein fractions from unfermented and semifermented-dry cacao seeds

Seed protein fraction	Unfermented seed		Semi-fermented dry seed	
	(mg/g AcDP)	(%)	(mg/g AcDP)	(%)
Albumin	33.00 ± 0.05	24.37 ± 0.10	66.15 ± 0.16 *	28.89 ± 0.01
Globulin	22.20 ± 0.01	16.39 ± 0.02	39.18 ± 0.08 *	17.11 ± 0.02
Prolamin	4.3 ± 0.13	3.21 ± 0.04	9.91 ± 0.16 *	4.33 ± 0.02
Glutelin	76.00 ± 0.13 **	56.02 ± 0.00	120.93 ± 0.04 *, **	52.81 ± 0.02

AcDP (acetone dry powder)

Data are expressed as the mean of the results of two experiments realized by duplicate ± S.E.M.

* *p *< 0.05 unfermented seed compared with semifermented dry seed.

** *p *< 0.05 when is comparing seed protein fraction regardless cacao process.

### Protein fractions characterization

**Figure 1:**
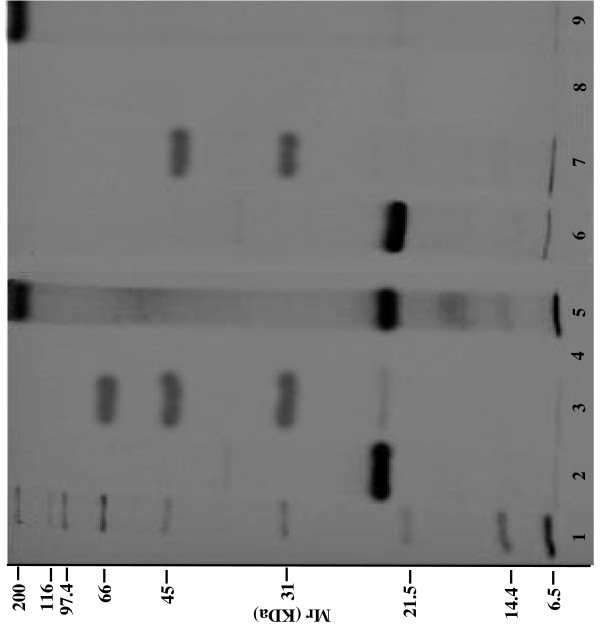
**SDS-PAGE profiles of cacao protein fractions (1: MW markers; 2: albumin UF; 3: globulin UF; 4: prolamin UF; 5: glutelin UF; 6: albumin SFD; 7: globulin SFD; 8: prolamin SFD; 9: glutelin SFD)**.

**Figure 2:**
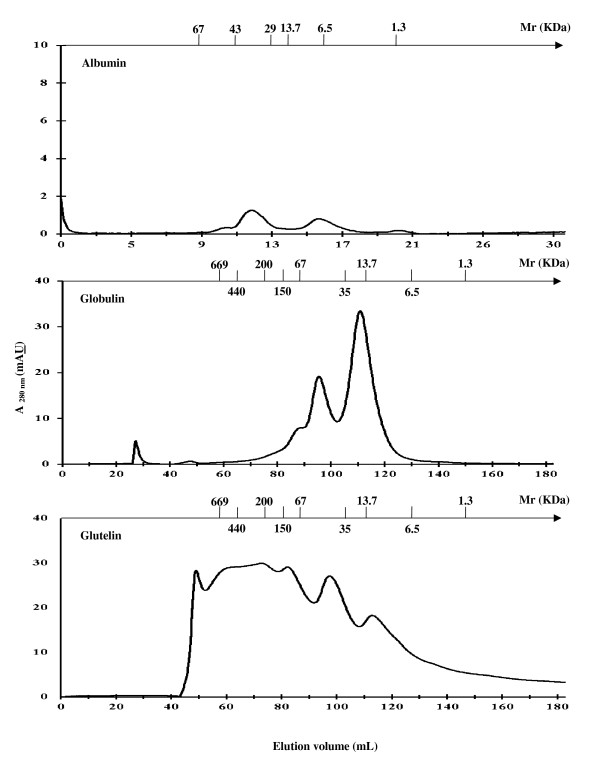
**Fractionation of unfermented cacao proteins by size exclusion chromatography (Albumin: Superdex G75 10/30 GL, equilibrium liquid and sample buffer 20 mM Tris-HCl, 150 mM NaCl, pH 7.2, flow rate 1 mL/min; globulin and glutelin: Superdex Hiload 200 16/60, equilibrium liquid and sample buffer 20 mM Tris-HCl, 150 mM NaCl, pH 7.2 and 20 mM potassium phosphate, 1.0 M NaCl containing 1% (v/v) NLS, respectively, flow rate 1 mL/min)**.

**Figure 3:**
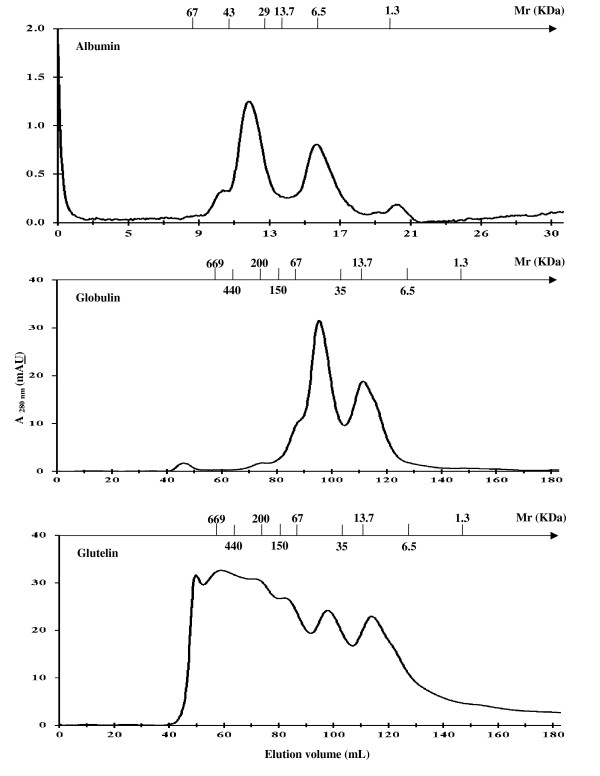
**Fractionation of semifermented-dry cacao protein by size exclusion chromatography (Albumin: Superdex G75 10/30 GL, equilibrium liquid and sample buffer 20 mM Tris-HCl, 150 mM NaCl, pH 7.2, flow rate 1 mL/min; globulin and glutelin: Superdex Hiload 200 16/60, equilibrium liquid and sample buffer 20 mM Tris-HCl, 150 mM NaCl, pH 7.2 and 20 mM potassium phosphate, 1.0 M NaCl, containing 1% (v/v) NLS, respectively, flow rate 1 mL/min)**.

The molecular weight and the molecular weight distribution of these protein profiles were determined by the one-dimensional electrophoresis (SDS-PAGE) technique (Figure 1) and analyzed by FPLC (Figure 2 and Figure 3). SDS-PAGE profile clearly showed that, in both cacao seed type, the albumin fraction had one predominant polypeptide of 21.5 kDa. The globulin fraction from UF cacao contained polypeptides with apparent molecular sizes of 66, 45 and 39 kDa, differing with the SFD cacao fraction that presented polypeptides of 45 and 39 kDa. Every polypeptide was detected in the prolamin fractions, in both types of cacao seeds. The polypeptide bands were similar to those obtained by several researchers for cacao beans in classical fermentation [26,37,38]. Glutelin fraction from UF cacao had proteins with molecular mass > 200 kDa and others of low molecular weight, 19.7 and 14.4 kDa, while the SFD cacao fraction presented only high molecular mass proteins (> 200 kDa) that were slightly higher than those reported previously [39].

A comparison was performed among the different FPLC profiles obtained using Superdex columns, which are molecular exclusion columns for the separation and purification of proteins. This comparison indicated that the albumin fraction was not significantly degraded during cacao fermentation, since two elution peaks were observed at a volume of 11 and 15.6 mL, being its estimated molecular mass 42.3 and 8.5 kDa in both cacao processes. These results indicate that albumin fractions in their native state could be composed of two peptide chains connected by non-covalent or covalent bonds; however native-PAGE electrophoresis and SDS-PAGE without β-mercaptoethanol were necessary for confirmation. This result was similar to those reported for amaranth, chickpea and lupin [40,41]. The globulin fraction was significantly degraded during cacao fermentation, observing the disappearance of elution peak at an elution volume of 88 mL and estimated molecular mass of 86 kDa, to produce the specific-cacao aroma precursors, which are polypeptides of 45 and 39 kDa. The elution peak with elution volume of 94 mL (~57 kDa) increased at the end of fermentation, as reported by several authors for classical fermentation processes [26,36,37,42]. Concerning the glutelin fraction, which was not degraded during cacao fermentation, it presented components of molecular weight > 200 kDa and globulin components of molecular weight < 100 KDa in a lesser proportion, such as the ones observed on SDS-PAGE electrophoresis, whose purpose has not been detected. However, the presence of a globulin fraction may be due to the fact that it has a better solubility than the glutelin fraction and therefore its concentration after sample filtration may be underestimated due to retention in the membrane.

These results did not agree with the work of Voigt and Biehl [26], who reported in unfermented and fermented seed yields of 52 and 43% for albumin and globulin fractions, respectively, without detecting a prolamin fraction. This authors stated that the glutelin fraction were residual globulins. In the present study, the difference may be partially attributed to geographic diversity, climatic conditions, and soil type, as well as the cacao benefit processing, which is quite different to the classical cacao fermentation.

### Antitumor activity of protein fractions

The antitumor effect of cacao protein fractions was evaluated using a murine lymphoma L5178Y model. The effect of cacao protein fractions on tumor growth is shown in Table 2. Preliminary assays demonstrated that the effect of proteins fractions (independent from the cacao process) on the model of murine lymphoma L5178Y was not dose-dependent, since after giving oral doses of 2.5, 25, 100 and 200 mg/kg/day the effects are oncostatics at low concentrations (<25 mg/kg/day) and induce significant tumor growth (p < 0.05) at high concentrations (>25 mg/kg/day) (data no shown). These results were consistent with those found for moderate protein deficiency, which did not enhance cellular immune responsiveness against melanoma in mice. It is noteworthy that high protein diets are suspected to be detrimental to the renal and hepatic functions, calcium balance and insulin sensitivity, such as increased urinary nitrogen excretion, glomerular filtration rate, kidney hypertrophy, renal hemodynamics and eicosanoid production in renal tubules. In addition, high protein diets caused increases in the levels of oxidative parameters, originating an unbalance between the production of ROS and the capacity of the antioxidant defense system of mice [43,44].

**Table 2:** Effect of the protein fractions from UF and SFD cacao Forastero seeds on body weight, tumor volume, packed cell volume, and viable tumor cell count of lymphoma L5178Y mice

Groups	Body weight (g)	Splenomegaly (g)	Tumor volume (mL)	Packed cell volume (mL)	Viable tumor cell count (×10^6 ^cells/mL)
Control L5178Y	30.83 ± 2.41	0.23 ± 0.05	2.67 ± 1.08	1.30 ± 0.49	412.62 ± 35.14
L5178Y + Globulin UF	29.32 ± 2.89	0.23 ± 0.11	3.00 ± 1.22	1.12 ± 0.87	286.96 ± 92.84
L5178Y + Glutelin UF	27.5 ± 1.73	0.19 ± 0.02	4.20 ± 0.64 *	1.97 ± 0.60	415.80 ± 92.88
L5178Y + Albumin SFD	28.08 ± 2.38	0.20 ± 0.07	0.60 ± 0.75 *	0.50 ± 0.30 *	750.00 ± 47.03 *
L5178Y + Glutelin SFD	31.06 ± 3.20	0.21 ± 0.01	3.50 ± 3.46	1.02 ± 0.95	365.10 ± 223.82

Non-lethal oral dose of 25 mg/Kg/day during 15 - 17 days, after inoculation of 6 × 10^4 ^lymphoma L5178Y cells/mL per mice

Data are expressed as the mean of the results of two experiments with n = 5 in each experiment ± S.E.M.

* *p *< 0.05 extract-treated groups compared with the lymphoma L5178Y control group

Body weight of normal mice: 23.50 ± 1.59 g. Spleen weight of normal mice: 0.12 ± 0.04 g.

The latter behavior was recurrent in UF cacao glutelin protein fraction, whose effect on tumor growth differed significantly (p < 0.05) as compared to the animals in the control group. It increased the ascitic fluid volume, tumor mass volume, and tumor cell count, which can be considered as an induction of cell growth in a 58.5 ± 11.9% in 100% of the population. However, the difference in the body and spleen weight of treated animals did not differ significantly (p > 0.05) when compared to the animal control group.

The best results were obtained by the SFD cacao albumin fraction, which had an effect on tumor growth that differed significantly (p < 0.05) to the other tested protein fractions and to the animal control group. It decreased the ascitic fluid volume, tumor mass volume, and tumor cells count, with a cell growth inhibition of 59.98 ± 13.6% in 60% of the population. However, the difference in body and spleen weight did not differ significantly (p > 0.05) to the one presented by the animal control group. These results suggested that such antitumor activity could be attributed to its particular amino acid profile, which has been described as rich in cysteine, leucine, arginine, and lysine, which relates to its trypsin inhibitor potential [37,45].

The results above indicate that there is no correlation between increasing body weight and the decrease in ascitic fluid, and also contradict that the increase in abdominal distension and body weight were a function of the increase in ascitic fluid during tumor growth [46]. In this regard, a study in BALB/c mice with protein-energy malnutrition, while undertaking the administration of formulas with hydrolyzed protein, found: 1) Increased nitrogen retention; 2) increased body weight gain; and 3) increased levels of total protein and branched amino acids in serum. This was similar to what was found by using intact proteins from different sources in the diet of Wistar rats. The assessment of body weight may be considered as one of the most widely studied factors in the detection of possible activity of a bioestimulant leading to the restoration of organism homeostasis [47,48].

### Antioxidant activity as free radical scavenging capacity of protein fractions

It has been reported that the different type of extracts (proteins, flavonoids, polyphenols or mixed) obtained from plants contain antioxidants, which present cytotoxicity against tumor cells and antitumor activity in experimental animals [46]. Three methods were used for measuring antioxidant activity: DPPH, TEAC and ORAC. The first assays where carry out by the DPPH system, according to Cheison *et al *[49] and Chevalier *et al *[50]. Solutions of protein fractions from UF and SF cacao were prepared at concentrations of 500 ppm up to 30,000 ppm. Precipitation problems were observed because the DPPH radical is dissolved in 80% (v/v) MeOH. The only exception was the prolamin fraction due to its intrinsic solubility in alcohols (data not shown).

Alternatively, the scavenging activity of the UF and SFD cacao protein fractions was determined by the ABTS* radical method. Trolox was used as a control sample and GHS for comparison purposes, given its high electron donating capacity (high negative redox potential) that generates a great intracellular reducing power [51]. Preliminary assays demonstrated that radical absorbance was stabilized after 30 min of reaction with samples or positive control, thus for comparison purposes 6 min of reaction was selected as the measurement time to obtain the scavenging% and the IC_50 _value from the corresponding plot. The highest scavenging capacity, expressed as TEAC, was presented for SFD cacao protein fractions (p < 0.05) achieving inhibition up to 80 - 90% at lower concentrations than UF cacao protein fractions. In both cacao processes, the glutelin fraction presented the higher scavenging capacity (p < 0.05) at any concentration assayed, with an IC_50 _value 7.1-fold higher than this of GHS (Table 3).

**Table 3:** Antioxidant activity of protein fractions from UF and SFD cacao seeds

Sample	Unfermented seed			Semi-fermented dry seed		
	ABTS^+ ^assay		ORAC assay	ABTS^+ ^assay		ORAC assay
	
	TEAC (mM Trolox/g protein)	IC_50 _(mg _protein_/mL)	TEAC (mM Trolox/g protein)	TEAC (mM Trolox/g protein)	IC_50 _(mg _protein_/mL)	TEAC (mM Trolox/g protein)
Albumin	0.14 ± 0.03 (3000)	3. 14 ± 0.08	0.44 ± 0.05 (70)	0.37 ± 0.01 (1500)*	1.51 ± 0.02*	0.65 ± 0.13 (50) *
Globulin	0.18 ± 0.03 (3000)	2.89 ± 0.08	0.17 ± 0.01 (300)	0.22 ± 0.04 (1500) *	2.21 ± 0.06*	0.29 ± 0.04 (150) *
Glutelin	0.88 ± 0.01 (600)**	0.58 ± 0.04**	1.21 ± 0.11 (70)**	1.32 ± 0.01 (300) *,**	0. 22 ± 0.02*,**	1.10 ± 0.73 (50) *,**
GHS	0.44 ± 0.11 (92)	0.081 ± 0.04	0.65 ± 0.44 (21)	0.44 ± 0.11 (92) *	0.081 ± 0.04*	0.65 ± 0.44 (21) *

TEAC (Trolox equivalent antioxidant capacity), GHS (Reduced glutathione).

Data are expressed as the mean of the results of two experiments realized by duplicate ± S.E.M.

Values in parentheses indicate protein concentration assayed expressed as ppm

* *p *< 0.05 unfermented seed compared with semifermented dry seed.

** *p *< 0.05 when is comparing seed protein fraction regardless cacao process.

To ensure that all the antioxidants present in the sample have reacted with the radicals generated at the end of the process, the ORAC method was assayed. It is one of the methods that combine the length of inhibition with the inhibition percentage of the free radical damage by antioxidants into a single quantity, both simple and sensitive [52,53]. Considering the decay of the fluorescence curve of FL, a homogeneous behavior was observed in the samples assayed at the highest concentration, thereby protecting FL from oxidation for at least 25 - 30 min, for both UF and SFD cacao. However, the best effect was shown by the glutelin fraction that increased the time of inhibition to 45 min at 70 and 50 ppm, for UF and SFD cacao seed respectively, surpassing the effect of 70 μM GHS (inhibition time 25 min) (data not shown). ORAC-FL values of total antioxidant activity, expressed as TEAC, in SFD cacao protein fractions showed a significant difference (p < 0.05) when compared to the values of UF cacao protein fractions. In both cacao processes, the glutelin fraction differed significantly (p < 0.05) from the albumin and globulin fractions (Table 3).

A significant linear correlation was found between radical scavenging capacity assays (0.68 ≤ r ≤ 0.97, p < 0.05) in both cacao processes (r ~ 0.90), despite the difference in the source of free radicals used in each method. TEAC and ORAC assays have been the most widely used electron transfer (ET) and hydrogen atom transfer (HAT) methods, respectively [34,54]. The TEAC or ABTS assay is based on scavenging of the ABTS^+ ^radical cation by the antioxidants present in a sample. In the reaction medium the antioxidant compounds capture the free radical, resulting in lost bluish-green color and therefore a reduction in absorbance, which is corresponded quantitatively to the concentration of antioxidant present in the sample [33]. The ORAC assay measures the ability of the antioxidant to protect the fluorescein from oxidative damage by a decrease in the fluorescence. The ORAC assay is said to be more relevant because it utilizes a biologically radical source of peroxyl radicals (ROO^.^) [34,52,55]. The ORAC assay has been used to study the antioxidant capacity of many food samples and has recently been adopted by the food industry as an adequate antioxidant index [56]. In this sense the cacao protein fractions may be considered as chain breaking antioxidant, which interrupt the chain reaction of radicals (propagation). The oxidative mechanism is the continued transfer of hydrogen atoms, forming a stable radical which does not continue the chain reaction or continues with low efficiency [55].

It is clear that SFD cacao protein fractions exhibited potent *in vitro *antioxidant activity in ABTS and ORAC free radical scavenging activity assays, allowing them to compete with other antioxidant compounds recognized in their pure state, such as ascorbic acid (1.05 TEAC), α-tocopherol (0.97 TEAC) and uric acid (1.01 TEAC) [57]. Therefore, SFD cacao seed still represents a significant source of antioxidants, since peptides and amino acids could be released during the cacao semifermentation-dry process, which may support their potential as a natural functional food. These results suggest that this antioxidant activity could be attributed to their constituent amino acids, which are capable of donating protons to free radicals, as well as to a probable formation of a hydrophobic oligopeptides feature during the first stage of fermentation. Although not a precursor of the cacao aroma, its hydrophobicity is an important factor to promote the availability of lipophilic oxidants which is expressed as a higher antioxidant activity [20,58].

### Correlation of antitumor effect in vivo with antioxidant activity in vitro

To our knowledge, this finding represents the first report of an inhibitory effect of cacao protein fractions on the model of murine lymphoma L5178Y. However, antioxidant activity *in vitro *of cacao protein fractions, in both cacao processes, did not present correlation with their antitumor effect *in vivo. *The biological system was more complex than the simple chemical mixtures assayed, and antioxidant compounds may operate via multiple mechanisms. These compounds were extensively metabolized *in vivo *and the antioxidant and biological activity of their peptides differed due to their particular amino acid sequences. Therefore, this model suggests that there are additional functions of cacao protein fractions as antitumor agents beyond their antioxidant capacity. Whether their antitumor effect was maintained after digestion, absorption and metabolism is unknown.

## Conclusion

Antitumor activity was only observed in the albumin fraction, which inhibited the growth of cells in murine lymphoma L5178Y. This could be attributed to its hydrophobic and sulfur amino acids profile that conferred antitumor and antioxidant potential. The antioxidant activity given by free radical scavenging capacity was observed mainly in the albumin and glutelin fractions from SFD cacao and it is considered as chain breaking antioxidant; however the highest values were observed for glutelins. Therefore, no direct correlation between antioxidant and antitumor activity was found. Protein fractionation showed albumins, globulins, and glutelins, even though the glutelin fraction had not been previously reported by researchers. This is the first report on the biological activity of semifermented-dry cacao protein fractions with their identification, supporting the traditional use of the plant. Further studies are underway, dealing with the evaluation of cytotoxicity and the elucidation of the action mechanisms, in order to considerer the protein fractions as a source of potential antitumor peptides.

## List of abbreviations

UF: unfermented cacao seed; SFD: semi fermented-dry cacao seed; AcDP: acetone dry powder; HCl: hydrochloric acid; EDTA: disodium dihydrogen ethylenediaminetetraacetate; NaCl: sodium chloride; NaOH: sodium hydroxide; BSA: bovine serum albumin; ABTS: 2,2'-azino-bis(3-ethylbenzthiazoline-6-sulphonic acid); Trolox: 6-hydroxy-2,5,7,8-tetramethychroma-6-sulphonic acid; PBS: phosphate buffer saline; TEAC: Trolox equivalent antioxidant capacity; APPH: 2,2'-azobis(2-methylpropionamidine) dihydrochloride; FL: fluorescein; GHS: reduced glutathione; DPPH: 2,2-Diphenyl-1-picryl-hydrazyl; ORAC-FL: oxygen radical antioxidant capacity assay using fluorescein.

## Competing interests

The authors declare that they have no competing interests.

## Authors' contributions

AMPYL carried out experimental work, data collection and interpretation, literature search and manuscript preparation. AMPP provided assistance in evaluating the antitumor activity and data interpretation. JCMD provided assistance in FPLC size-exclusion chromatography and data interpretation. RHG provided assistance in SDS-PAGE gel electrophoresis and data interpretation. MEJF and ECLC supervised the work, evaluated the data and corrected the manuscript for publication. All authors read and approved the final manuscript.

## Pre-publication history

The pre-publication history for this paper can be accessed here:

http://www.biomedcentral.com/1472-6882/10/61/prepub
